# Jobst-Hendrik Schultz, Simone Alvarez, Christoph Nikendei: Heidelberger Standardgespräche: Handlungsanweisungen zur ärztlichen Gesprächsführung mit zahlreichen kommentierten Filmbeispielen

**DOI:** 10.3205/zma001275

**Published:** 2019-11-15

**Authors:** Swetlana Philipp

**Affiliations:** 1 Friedrich-Schiller-University Jena, University Hospital Jena, Institute for Psychosocial Medicine and Psychotherapy, Jena, Germany

## Bibliographical details

Jobst-Hendrik Schultz, Simone Alvarez, Christoph Nikendei

**Heidelberger Standardgespräche: Handlungsanweisungen zur ärztlichen Gesprächsführung mit zahlreichen kommentierten Filmbeispielen**

Publisher: HeiCuMed, Heidelberg University Medical Faculty

Year of publication: 2018, pages: 244, prize: 29,99 €

## Recension

With this book, the authors have produced a pocket guide that can be of use to students as well as teachers and standardized patients. The “Heidelberg Standard Consultations” cover doctor-patient communication in both human medicine and dentistry.

The first part of the book is devoted to a general overview of medical consultation skills and describes numerous models of communication. Amongst other, the theories of Watzlawick, Schulz of Thun, Berne, Prochaska and Langewitz and models such as WWSZ, CALM, SBAR, NURSE and SPIKES are introduced over approx. 25 pages. This gives both students and lecturers a good overview of the special aspects of the different types of consultation and communication techniques. But even standardized patients can benefit from awareness of the theories in this pocket guide, providing useful background information for role-playing and giving feedback. The advantage is that all three target groups of this book (students, lecturers and standardized patients) can then analyze and understand the discussions between doctors and patients using a common vocabulary. 

In the second and most comprehensive part of the book, human medical consultation situations from the pre-clinical (6 cases) and the clinical (33 cases) phase of the Heidelberg communication curriculum HeiDuMed are described, as well as two examples of ward round communication. Each individual case is structured in such a way that the reader first gets to familiarize themselves with the case profile and task. Then, 2 to 4 learning objectives are formulated with appropriate references to the National Competence-Based Catalog of Learning Objectives. Subsequently, the reader learns more about the background, medical history, biography and social situation as well as case-specific characteristics of the role of the standardized patient. 

For about half of the presented case studies, video clips were recorded and uploaded to the book’s homepage for the buyers to view. Some clips are also available for the doctor-patient consultations, both successful and less successful consultations, allowing critical examination of the different role models. The variety of topics ranges from a “taking the patient history of a hearing-impaired patient”, “taking the patient history from a migrant patient” to “information and consultation session with a patient with analgesic-induced headaches” (for the pre-clinical phase) to “confronting an anorexic patient with their condition”, “talking to a relative of a geriatric patient” and “consultation with a married couple suspected of domestic violence“ (clinical phase). The two variants of the following theme are also very interesting: “Referring a patient with exacerbated chronic obstructive pulmonary disease to nursing care”, which showcase impressive examples of the difficulties of interprofessional communication. All in all, a very broad spectrum of consultation situations and communicative tasks are covered. 

The video clips of two variants of taking the history of a depressed patient (commented and uncommented) can be freely accessed at https://www.heidelbergerklinischestandards.de/buecher-videos/sign-in-gespraeche/. 

In the third part of the pocket guide, four dentistry consultation situations from the HeiDuDent are presented. These include taking the history of a diabetic woman with chronic periodontitis, a patient with iatrogenic nerve damage, surgical wisdom tooth removal, dental anxiety; and a patient with somatoform disorder. 

The fourth part of the book contains the chapter “Communicating risky procedures” where we learn about the particular challenges in doctor-patient communication in the context of participatory decision-making, for example discussing and assessing mammography diagnostics; and “Mentalizing in medical consultation” with very helpful phrases for effective mentalization. 

The fifth and final chapter contains 12 guides for challenging situations, which are then also linked to the topics of the case studies or videos. So the first section tackles “Dealing with hearing-impaired patients” (see above), the second with the topic “Intercultural communication” and the last “Dealing with treatment anxiety”.

### Conclusion 

From my perspective as a lecturer in medical psychology where I teach doctor-patient communication and train the standardized patients at University Hospital Jena, I would recommend this book as very stimulating and helpful. Case studies that are relevant for students, lecturers and standardized patients have been presented in an easy-to-understand manner and are backed up with valuable additional material. 

Overall, the pocket guide constitutes a clear and usable handbook about medical consultations with standardized patients. 

## Competing interests

The author declares that she has no competing interests.

